# Air conditioning and the future electricity system in Great Britain

**DOI:** 10.1016/j.erss.2026.104727

**Published:** 2026-06

**Authors:** Charles H. Simpson, Sapna Halai, James Price, Giorgos Petrou, Clare Heaviside, Michael Davies

**Affiliations:** a Bartlett School of Environment, Energy, and Resources, University College London, Central House, 14 Upper Woburn Place, London, United Kingdom

**Keywords:** Air conditioning, Renewable energy, Electricity system, Future energy system

## Abstract

Climate change is driving hotter summers in temperate regions including the United Kingdom, where historically air-conditioning demand has been low and energy-system planning focused on winter peaks. As Great Britain’s energy system moves toward renewables-dominated supply, understanding how emerging cooling demand could interact with system adequacy becomes increasingly important.

Here, we use a risk-opportunity analysis framework to examine how cooling uptake could affect future planning and adequacy of the electricity system, integrating international ownership data, qualitative analysis of policy, and first principles system-capacity calculations. We aim to identify where cooling creates underexplored risks or opportunities, and what assumptions need revision.

We find current official scenario assumptions for residential air conditioning uptake are too narrow, and present ownership is potentially an order of magnitude higher; therefore, the system-level implications of more rapid cooling uptake remain essentially unexplored. While there may be a noon surplus of solar energy in summer, official scenario analysis does not yet examine peak loads in summer and may therefore miss interactions with low-wind periods and declining afternoon solar power, potentially underestimating requirements for storage and flexibility.

We recommend summer adequacy analysis be included in planning. Underestimated cooling demand could lead to missed decarbonisation targets or greater operational challenges, but treating low demand assumptions as constraints could lead to missed policy opportunities to protect health from overheating and to make use of reversible heat pumps. Air conditioning needs to be understood within a systemic context of electrification of heating, expansion of renewable energy, and adaptation to higher temperatures.

## 1 Introduction

Use of air conditioning is expected to grow globally due to rising incomes, expanding access to electricity supply, falling equipment prices, and increasing temperatures due to climate change [1]. Although temperate countries have, historically, had low demand for residential space cooling relative to demand for space heating, climate change is increasing the frequency and intensity of extreme heat events which may drive greater space cooling demand [2]. In temperate countries, annual peak electricity demand has typically occurred in winter, so energy system planning has focussed on winter adequacy.

Growing electricity demand from air conditioning is often framed as a problem, for example, an IEA report stated that “[a] concerted policy push to rein in cooling energy demand is needed urgently” [3]. In hotter countries, air conditioning contributes strongly to peak electric power demand and total energy consumption. Supplying peaks in power demand is often expensive [4] and can lead to greater fossil fuel usage [1,3,5]. Capacity in electricity generation, transmission and distribution infrastructure can also be reduced in hot weather, which in combination with high demand can lead to load shedding or increased system costs [1,6,7]. However, many countries already have or are planning lowcarbon electricity systems that deliver much higher peak loads to meet demand for electric space heating: such systems will be adequate for some amount of space cooling load. As we will argue, the additional effect of rising space cooling demand in systems dominated by heating demand is currently not well understood.

These challenges are particularly evident in the Great Britain (GB). (GB includes England, Scotland and Wales. Northern Ireland is served by a separate electricity system) Due to climate change, summers in GB are expected to become hotter and drier, with heatwaves becoming more frequent and intense [8]. England experienced ambient temperatures exceeding 40 °C for the first time in July 2022 [9]. With 2 °C of global warming the ratio of cooling degree days (degree days above 22 °C) to heating degree days (degree days below 15.5 °C) in England is projected to increase to 1/27 from its current 1/85 [10], and just 1/13 in London.

GB is covered by the UK’s legally binding net-zero greenhouse gas emission targets and has detailed scenario modelling through the Future Energy Scenarios (FES) produced by the National Energy System Operator (NESO). These scenarios are the basis for electricity system planning, and generally assume that growth in residential cooling will either remain low or occur only in later decades (2040s onwards). Total demand for cooling will likely remain smaller than total demand for heating, and as a result NESO has argued that cooling demand will not be challenging [11]. NESO has argued that solar electricity generation will be well placed to meet space cooling demand because they are driven by similar weather [11]. However, we highlight several problems with these assumptions that reflect how cooling is overlooked in GB energy policy more widely. The FES 2024 assumptions about future residential cooling uptake are overly narrow, and recent surveys suggest that residential cooling ownership may be growing far more rapidly than assumed. Furthermore, quantification of peak demands from residential cooling have not been incorporated into adequacy analysis for the FES. This suggests that residential space cooling could be a blind spot in energy system design in GB.

Yet, if the electricity system challenges are small or can be overcome, then residential cooling could present a synergy between climate mitigation and adaptation policy: an opportunity to improve comfort and productivity, protect health from heatwaves, and to incentivise uptake of heat pumps to replace natural gas for heating.

The consequences for the energy system of growing residential air conditioning demand in temperate countries represent an important but under-examined risk for energy system planning. In this article, we aim to examine the emerging risks and opportunities presented by the growth of air conditioning in GB. We use a mix of qualitative and quantitative methods, drawing on policy documents and official statistics where appropriate, and use a narrative structure to evaluate how different trajectories of cooling uptake could interact with energy policy. We review relevant policy documents including the Future Energy Scenarios [12] to identify how air conditioning demand assumptions may be influencing policy and planning, as well as the documents and studies that have been used to support their assumptions especially those which have made quantitative future projections (such as the Cooling in the UK report [13]). We also compare air conditioning ownership data from other countries in Europe. We argue for a scenario-based risk-opportunity analysis framework rather than relying on a single forecast trajectory [14]. While focussed on GB, our analysis has broader relevance for other temperate countries facing uncertain but growing residential cooling demand. Overall, we aim to identify where rapid changes in cooling demand could create underexamined risks, where they may offer synergistic opportunities, and where planning assumptions need revision.

This paper makes four novel contributions. First, we compile evidence showing that residential air conditioning ownership is likely higher and growing faster than assumed in official GB energy planning scenarios. Second, we identify that FES analyses omit summer adequacy and demand-net-of-renewables dynamics, and that these will become relevant as cooling expands. Third, we identify drivers that make rapid uptake plausible despite past expectations of slow growth. Finally, we situate cooling in a risk-opportunity framework that recognises its transformative potential allowing synergy between climate mitigation and adaptation. Together, these findings identify cooling as a blind spot in energy planning in GB.

The article is structured as follows. In Section 2, we outline the policy context of planned changes in the electricity system in GB. In Section 3, we review the evidence that air conditioning ownership is growing rapidly, and review policy documents to identify how future projections have been used to inform policy. We consider how the varying drivers of growth in air conditioning ownership may make these projections misleading. In Section 4, we consider whether this growth in demand represents a risk to the adequacy of the electricity system in GB through quantitative comparisons of load and capacity. In Section 5, we consider to what extent an expanded role for air conditioning represents an opportunity for energy and climate policy if energy system challenges can be met, qualitatively relating these different areas of policy with a system-wide view. Section 6 summarises the results and draws overall conclusions.

## 2 Policy context

Policy research on decarbonisation of space cooling often refers to some variation of an approach called “avoid, improve, shift” adapted from transport sustainability policy (e.g. [15–17]). In this context, “avoid” means reducing the need for air conditioning, “improve” means using standards to ensure efficiency and phase out of high-GWP refrigerants, and “shift” means using zero-carbon energy. Examples of “avoid, improve, shift” can be seen in UK policy. Recent additions to the building regulations require new buildings to address overheating, and suggest “using passive means as far as reasonably practicable” with air-conditioning only used if the requirement cannot be met “using openings”, an example of “avoid” [18]. Policies which focus on improving efficiency standards for air conditioning or reducing the global-warming potential of refrigerants, (e.g. [19]) are examples of “improve”. Hot weather is often correlated with solar irradiance in the UK, so there is the potential to meet demand for energy for air conditioning through solar power, storage, and flexibility [11,16], an example of “shift”.

Big shifts are planned in electricity systems in the UK. The UK has a legal commitment to reach net-zero greenhouse-gas emissions from the whole economy by 2050. As part of this transition, targets for Great Britain’s electricity system are for “Clean sources [to] produce at least as much power as Great Britain consumes in total [and …] at least 95% of Great Britain’s generation” by 2030 [20], which is expected to reduce the emissions intensity of electricity generation to “well below” 50 gCO_2_e/kWh Over the longer term, meeting net-zero emissions will require emissions intensity close to zero: The Climate Change Committee’s advice for the 7th Carbon Budget models emissions intensity for electricity generation of just 1 gCO_2_e/kWh in 2050 [21].

The UK Government’s Clean Power 2030 Action Plan (“2030 Plan”) (which is aligned with the 6th Carbon Budget), paints a picture of a future energy system in GB in which most demand will be met by renewables (mainly wind and solar power). Decarbonisation of electricity is to be achieved mainly by increasing generation capacity for wind and solar photovoltaic power, as these are the most cost-effective forms of low carbon generation and have a proven track record of being deployed at scale. In the plan, positive demand net of renewables (DNR), i.e. power demand exceeding the power supplied by renewables, will be met by flexible power such as batteries, low carbon dispatchable power such as hydrogen, but also with unabated natural-gas generation to ensure security of supply [20,22]. If fossil fuel generation is only used when there is positive DNR, then the emissions intensity of electricity will be highly variable in time. Energy used at times of sufficient renewable generation would have zero emissions, whereas energy used at times of positive DNR may have a greater marginal effect on fossil fuel use [23].

If this substantial “shift” in the electricity system is achieved, an important question arises: how much effort should be directed toward “avoiding” and “improving” air conditioning? The electricity sector and domestic buildings must be considered as an integrated system rather than as separate policy problems. In a future energy system in which emissions intensity of generation varies strongly through the year and day, reducing demand at times of peak DNR will become much more impactful than reducing demand in general, which may complicate existing approaches to energy efficient building design [23]. In practice, any plausible future scenario will involve a combination of passive and active cooling strategies. However, there is currently limited understanding of how additional residential cooling demand would affect the future energy system, whether in terms of infrastructure costs, operational emissions, or system adequacy. Without a clear quantification of how much demand reduction is necessary, it is difficult to assess the value of interventions aimed at limiting cooling uptake; if demand would be low anyway, then measures to reduce it may be unnecessary. Measures to increase energy efficiency or reduce demand are not necessarily more cost effective compared to expanding system capacity (e.g. adding solar generation), and will have less effect on emissions as the transition to a low-carbon electricity system progresses [23]. On the other hand, if (for example) additional flexible generation is required then this could contribute substantially to system costs. Without a clearer understanding of the system-level consequences of increased cooling demand, it is difficult to determine the most appropriate balance of strategies.

## 3 Evidence that cooling demand is already diverging from official assumptions

Demand for power by domestic air conditioning will depend upon how many air conditioning units there are, as well as how much they are used; both of which are highly uncertain. In this section we examine estimates of current trends in domestic air conditioning ownership in the UK (Section 3.1). We then examine how different studies have produced projections of future use of air conditioning (Section 3.2), and what could be driving changes in air conditioning use (Section 3.3).

### 3.1 Surveys of air conditioning ownership

Estimates of present-day air conditioning ownership vary widely (Table 1). These surveys were conducted in different ways so may not be fully comparable, but there is a trend that more recent surveys give larger estimates. Two recent surveys (2023–2024) give much higher estimates ranging from 8 to 19% of households, whereas older surveys give lower figures, around 2%. The most recent official data published was collected May 2021 to March 2022 and found 3.1% of households reported using air conditioning [24]. Notably, one recent survey found that, in 2023, 81% of households with an air conditioner had purchased it in the past two years, which suggests that air conditioner ownership may be growing rapidly.

**Fig. 1:**
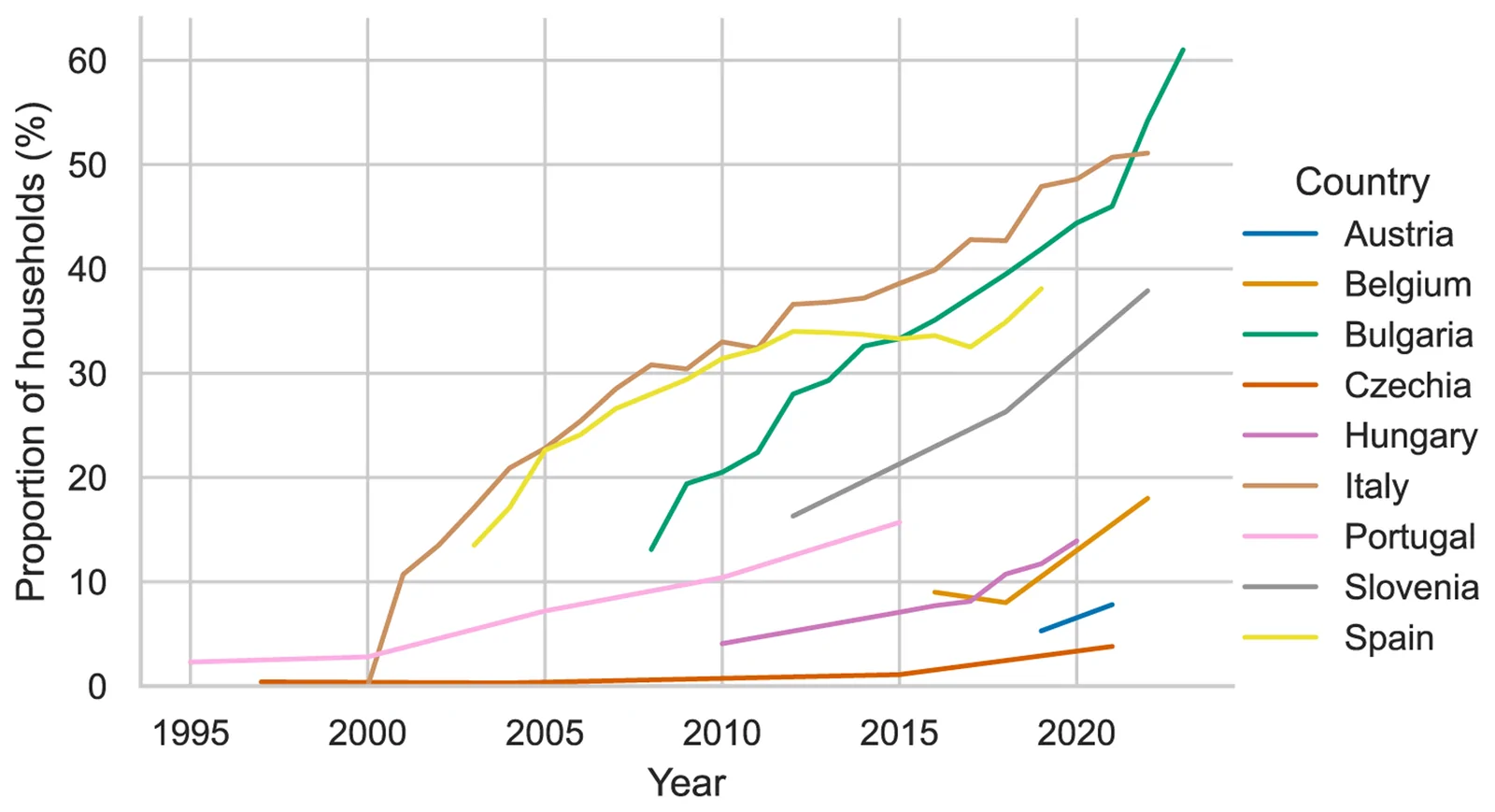
European countries generally report increasing trends in air conditioning ownership. Proportion of households owning any air conditioner as reported in national-level primary data, compared to recent UK surveys. Sources in Table 2.

Other European countries that report primary microcensus data on ownership of air conditioning generally show increasing trends (Fig. 1). The country which releases detailed data of this type, and is most similar to the UK in terms of climate and economy is Belgium, which reported that 11% of households have a portable air conditioner and 7% a fixed air conditioner in 2022, up from 8% and 5% respectively in 2020 [27].

### 3.2 Future projections of air conditioning ownership

Estimates of future growth rates of air conditioning in the UK have relied on data of the adoption of air conditioning in other countries, either by using annual growth rates in air conditioning ownership from other countries’ historic data (a time series approach), or looking at differences in air conditioning between places (a cross-sectional approach).

A frequently cited example of the cross-sectional approach is Sailor and Pavlova [28], which estimated an exponential relationship between annual average cooling degree-days (CDDs) and air conditioner ownership across states of the USA (CDDs are a metric commonly used to estimate how ambient temperatures affect cooling demand). However, an influential report “Cooling in the UK” commissioned by the UK Department of Business, Energy and Industrial Strategy (BEIS) noted that the Sailor and Pavlova [28] relationship predicted air conditioning ownership of 28% for London in 2020, well above their estimate of 5%, so BEIS made ad-hoc adjustments to create their own future projections for the UK [13]. Projected changes of average CDD from 2020 to 2030 are modest, so this method produces small absolute growth in peak electricity demand from air conditioning up to 2030: from a baseline of 11.3 GW peak demand in 2020 to 11.9 GW in 2030 (this includes non-residential buildings). In this model, most growth in air conditioning demand occurs after 2050, with peak demand reaching 19 GW in 2100 with 4 °C of warming. Regression of ownership across states of the USA against CDD has also been used by other authors to predict future air conditioning ownership [29], which has been cited in policy documents [30,31]. Others have made more ad-hoc assumptions about the relationship between CDD and air conditioning ownership [32].

A report by the Tyndall Centre commissioned by Electricity North West Ltd. (ENWL) took a time series approach based on annual air conditioning ownership in the USA, Australia, and France (summarised in Table 2) [33]. The report emphasised lower growth rates estimated from the USA and Australia, rather than the higher growth rate observed in France, without a clear justification for doing so. The results implied that it would take air conditioning ownership 15–17 years to double, which is contradicted by the data from France which implies just 3 years to double (note that they assumed an exponential growth model). As a result, their estimates of near-term growth in air conditioning were small in absolute terms when applied to the low starting level of UK air conditioning ownership. Newer data from other European countries also imply higher rates of growth under the same exponential assumption (Table 2), although the growth curves in Fig. 1 suggests exponential growth may be a poor model.

Overall, both of these influential reports (The Tyndall Centre report [33] and BEIS [13] report) predicted very small absolute growth in air conditioning ownership in the short term. In the meantime, air conditioning ownership appears to be growing faster than either of these reports predicted.

There could be methodological reasons why estimates based on past data in other countries do not transfer well. The assumption of exponential growth may not be justified. Also, past changes over time may be misleading as the increase in air conditioning in the past was due to changes such as market development, rising incomes, and changes to building practices rather than a changing climate [33]. Furthermore, differences in air conditioning ownership across places are not necessarily comparable to changes in ownership in time: buildings in different places may be constructed with local climate in mind and so already have some passive adaptations, whereas the climate may now be changing faster than the building stock is being adapted. Assuming that changes in air conditioning ownership are driven by changes in longterm average temperatures or CDDs may falsely lead to the conclusion that changes will be relatively slow. These structural assumptions are highly influential on the projected outcome and are not usually closely examined.

### 3.3 Drivers of rapid uptake

Another potential oversight is that international comparisons do not necessarily relate to the same technologies. For example, in Australia and the USA central systems using air ducting are popular, and are usually installed into new buildings rather than added to existing ones [33]: this has led some (e.g. [33,42,43]) to argue that growth in air conditioning in the UK is likely to be slow because homes are unlikely to install air ducting. However, split air conditioning systems (rather than ducted systems) are more common in the UK and Europe, and their installation is less intrusive [44]. Furthermore, British homes have drastically changed their services in the past 50 years for comfort: central heating requires extensive pipework installation throughout the entire home but was still retrofitted to many homes [45], so pipework may not be a long-term barrier if households are motivated to improve their comfort. There are also interactions with other services, for example adoption of heat pumps that can provide both heating and cooling has led to a level of access to residential cooling in Norway, Finland, and Sweden that may be surprising given their climates [46].

An important factor in rising air conditioning ownership may be portable air conditioners, which already make up the majority of the domestic air conditioner market in the UK [13,26]. Portable air conditioners are plug-in devices requiring no installation, so represent a straightforward consumer purchase rather than an intrusive change to a building. Portable systems also have lower purchase prices than fixed systems, and it is often suggested that such portable units are bought reactively after experiences of overheating [13,47]. This would suggest that ownership of air conditioning could grow with the frequency and intensity of heatwaves driven by climate change, and more quickly than indicated by past data from other countries. Portable air conditioners are less efficient than split systems, and may be used differently, so could produce quite different patterns of electricity use.

Overheating of residential buildings is a growing concern in the UK, with surveys indicating that a substantial proportion of households experience uncomfortably hot indoor conditions during the summer. For example, the 2023 English Housing Survey found that 12% of households reported at least one part of their home got uncomfortably hot [48]. The Energy Follow Up Survey conducted in 2017 found 17% of households reported that a bedroom in their home was uncomfortably warm often or all the time. [49] Another survey conducted in 2023 found that 80% of respondents reported that they had difficulty keeping comfortably cool in at least one room of their home [47].

Hot weather has well documented effects on mortality rates, although the effect of indoor overheating on mortality is less well studied. Without adaptation, the rate of heat-related mortality could increase by a factor of six from 2.5 to 15.1 per 100 k population by 2050, driven by increasing ambient temperatures [50]. There is a strong public health case for addressing overheating. Air conditioning is protective against heat-related mortality [51–53] for those who have access and can afford the operating costs [54].

Energy for heating and cooling are discussed in quite different ways in the UK currently. Cooling is seen as an “excessive and indulgent” luxury by many [25], whereas heating is seen as a necessity: this makes a lot of sense given the UK’s past climate. It is even sometimes suggested that air conditioning might need to be restricted to those who need it most (e.g. [42]). Energy for heating is regulated and subsidised because its role in comfort and health, especially that of older people, is widely recognised. However, this wasn’t always the case: central heating only grew in popularity in the 1970s [45], before this it was common practice to heat only one room [55], and typical temperatures in homes during winter were 13 °C [56]. Will perceptions of cooling energy change with the changing climate? Two-thirds of respondents to a recent survey “agreed that air conditioning is becoming a necessary addition to UK homes due to rising summer temperatures caused by climate change” [25]. Protecting health from the effects of climate change could mean adding air conditioning to more buildings, or even government support for some vulnerable people to use air conditioning.

While UK policy on overheating emphasises a “passive first” approach which can be seen in planning policy and in building regulations, there is not yet evidence that these regulations in practice are producing buildings that overheat less. It is also possible that the requirement from the building regulations to address overheating could overall increase the installation of air conditioning despite its “passive first” framing [57]. Furthermore, these regulations apply only to new buildings and there is no equivalent regulation or policy addressing overheating in existing buildings.

In some buildings, air conditioning may be the most cost-effective choice for reducing overheating. BEIS reported that portable air conditioning was cheaper (in terms of capital cost) and more effective than many modelled passive strategies including various external window shading options and internal blinds [13]. There are often barriers to implementation of proposed passive solutions in practice, for example many existing buildings are not suitable for external shutters (which can be very effective in principle) [57]. While the running costs may be substantial, lower upfront costs will be more important for many households, and so portable air conditioning may be the default response to overheating.

These trends challenge the assumption that residential cooling demand will remain small or grow slowly in the UK. Growth is not only driven by rising temperatures, but also by the availability of low-cost units that bypass some of the barriers associated with fixed installations, and changes in regulations and building practices. The limitations of passive cooling strategies, and especially the barriers for existing buildings, may mean that air conditioning is seen as necessary, or a default option by many households or developers.

Several studies (Section 3.2) have made predictions about the future trajectory of air conditioning use in the UK. While these predictions are uncertain, we think there may be limited value in resolving prediction uncertainty in relation to exogenous drivers such as ambient temperature, as there is an irreducible uncertainty driven by changing norms, attitudes, behaviours, and regulations. Furthermore, looking at the variations in air conditioning ownership between locations with different climates, or how quickly air conditioning has grown in other countries, can help identify what change is possible, but may be misleading because of local differences in technology and practices. It may be more important to understand the consequences of policy choices, and how these produce risks and opportunities [14], rather than trying to predict the most likely outcome based solely on exogenous drivers. It is also necessary to recognise the connection between different policy choices and sectors [58]: in this case, how choices in climate adaptation policy are influenced by, but also influence, choices in the energy supply and heating decarbonisation policies.

## 4 Implications for summer adequacy in renewable energy systems of underestimated cooling

In this section, we explore whether rising use of air conditioning presents a risk to the electricity system in GB, in the context of a rapidly decarbonising electricity supply and electrification of end-uses. We begin by discussing demand net of renewable energy in the future energy system (Section 4.1), before examining the adequacy of power supply (Section 4.2) and distribution (Section 4.3) for air conditioning demands in GB.

### 4.1 Demand net of renewable energy

The FES focus on having enough generation capacity to meet peak demand in winter, which is expected to be more challenging than summer. NESO stated “Much of our current modelling focuses on whether GB [will] have enough generation capacity to meet demand based on a peak demand occurring in winter. This is due to winter peak demand posing more challenges than in the summer. We are confident there will be sufficient supply to meet electricity demands over the summer and we will be able to meet operability challenges” [11]. However, this should be considered in the context that gas generation will be on standby: NESO is stating that there is not a substantial risk of power shortages, load shedding etc. caused by a lack of generation, rather than that there would be enough renewable generation [22]. FES pathways suggest unabated natural-gas generation will be phased out sometime between 2035 and 2050.

As weather dependent renewables (especially wind and solar) become a larger share of generation, challenging periods for the system shift from being times of maximum demand to maximum DNR [59–61]. In models of GB’s energy system, as increasing amounts of wind power capacity are added, the most challenging days shift from being the coldest days (which currently have the highest demand) to days with the combination of cold temperatures and low windspeed [59–63]. Solar power is less relevant for meeting these winter peaks in demand, due to solar angles and lack of clear skies. The energy system must be designed to be resilient to extremes of weather such as long periods of low wind generation called ‘wind droughts’ [30,64]. Due to computational constraints, the FES pathways are optimised using a single historical year’s weather but are tested for adequacy against years with long periods of low wind, finding that reliability would be similar to present but that the clean power targets might not be met in years with low wind [22].

Summer positive DNR events (which might be driven by high demand for cooling) have been studied in less detail than winter positive DNR in the UK [65]. Partly this is because it is assumed that demand from electrified heating will be very large compared to demand from air conditioning (cooling) in future scenarios for the UK. For example, BEIS estimated peak electricity consumption for space cooling (including domestic and non-domestic buildings) would be 19 GW in a scenario with low efficiency and high global warming (4 °C of global warming in 2100), compared to around 150 GW for heating in the same scenario [13]. Substantial investment in and improvements to the electricity system are required to meet the challenges of electrified heating, but if correct, the relative scale of the loads would imply that this improved infrastructure may also be sufficient to support an expansion of cooling. However, we argue that this could be an underestimate of the potential cooling load, for the reasons outlined in Section 3.

Furthermore, summer temperatures are often highest in the UK during clear-sky, low-wind conditions; wind power generation would be low but solar power generation would be high. While hot days are usually sunny days in the UK, the timing of cooling demand could be important: for example, air conditioning demand at night or in cloudy weather would not coincide with solar output [11,66]. NESO stated “solar [photovoltaic] output will likely correlate with [air conditioning] loads”, and while air conditioning demand at night would not correlate with solar power output, only a “few hours’” flexibility (such as “batteries and smart electric vehicle charging”) would be required to manage this [11]. Hence, if air conditioning does coincide with peaks in solar generation, then it may not contribute strongly to greenhouse gas emissions, to requirements for firm generation, flexible generation or storage, or to marginal costs. These arguments suggest that demand from residential air conditioning in GB will not be a problem for the electricity system. But, as we discuss below, this conclusion rests on questionable foundations.

Air conditioning is not mentioned in the NESO advice for the 2030 Plan, but it is based on NESO’s Holistic Transition (HT) pathway, their lowest electricity demand scenario (in the 2024 FES) [22]. In HT, residential air conditioning ownership is unchanged at 1% of households until 2050 (pointing to the BEIS “Cooling In The UK” report to support this [13,67,68]) [69]. The 2024 FES also presents other scenarios in which air conditioning ownership is greater in 2050, but all starting from 1% of homes in 2023, ranging between 10 million and 18 million homes with air conditioning in 2050 [70]. While there is substantial spread between the assumptions in 2050, changes are assumed to be exponential in time, meaning that absolute changes by 2030 are small and most change occurs in later decades: in these scenarios electricity demand for residential air conditioning increases by a factor of, at most, 2.8 from a low baseline. However, evidence we have presented (Section 3) shows that air conditioning ownership may already be higher than assumed by all the NESO FES 2024 scenarios for 2030, and (some surveys suggest) higher by an order of magnitude than assumed in the HT FES on which the Clean Power 2030 plan is based. This implies that the demand trajectories in FES and underpinning major policy may already be invalidated and obsolete.

Peak demand estimates from air conditioning are not presented in the FES 2024 workbooks. The assumption used to estimate the energy consumption of portable air conditioners in the FES is that each unit has a maximum power consumption of 2.7 kW, but that on a diversified basis (i.e. not all at full power at the same time) this creates an average consumption of 1 kW per unit in the main cooling period [71]. With an average of one unit per household, this would contribute around 30 GW to peak load. This assumption should also be treated as uncertain, and others have assumed higher power per household, e.g. 1.7 kW in Kingsborough et al. [32]

In summary, the FES pathways show zero-carbon generation in the summer in 2030, but the effect of residential air conditioning on this is not well explored. In our opinion the assumptions about residential air conditioning ownership by 2030 are unrealistically low in all the FES 2024 pathways, and it is a problem that variations between the pathways only appear in later decades. Furthermore, peak demands from residential air conditioning do not feature in the discussion of FES scenario design. This may be because the scenarios are deliberately optimistic about energy demand or because winter peaks are seen as more important, but as a result it is unclear if residential cooling demand needs to be low for the scenarios to be achievable.

### 4.2 Generation capacity

Rather than trying to predict the future trajectory of residential air conditioning ownership and peak demand, an alternative question might be: how large would air conditioning ownership need to be for it to create positive DNR in future pathways? To use this framing adopts a risk analysis approach, by asking what the situation is that needs to be avoided, rather than what the most likely outcome is.

A starting point is to consider the capacity factors of variable renewables. It makes sense to focus on solar electricity generation rather than wind, as hot summer conditions in the UK usually have clear skies and low wind speed. Daily capacity factors for solar generation will be higher than the annual average in these conditions, so it is reasonable to assume that air conditioning usage will be correlated with solar generation at the daily level (as assumed by [11,16]). However, domestic air conditioners are likely to be run in the evening or night when most people are at home [66]: a survey commissioned by ENWL found that “getting a good night’s sleep” was the most commonly mentioned motivation for purchasing an air conditioning unit [25]. Therefore, the hourly profiles of generation and demand will need to be considered, as this may affect requirements for storage and flexibility.

In California on 14–15 August 2020, the combination of an extreme heatwave (historically a 1-in-30-year event, but made more likely by climate change) alongside shortcomings in market, planning, and adequacy processes led to a shortfall in supply; as a result controlled load shedding was used to restore operating margin [7]. The extremely hot weather led to high electricity demand from air conditioning, but the resulting emergency was caused by multiple simultaneous operational stresses. Notably, the shortfall did not occur at the time of maximum demand, but later in the afternoon when DNR peaked due to demand remaining high while solar supply declined. This demonstrates the importance of hourly-scale DNR dynamics in systems with high penetration of solar power and air conditioning.

**Fig. 2:**
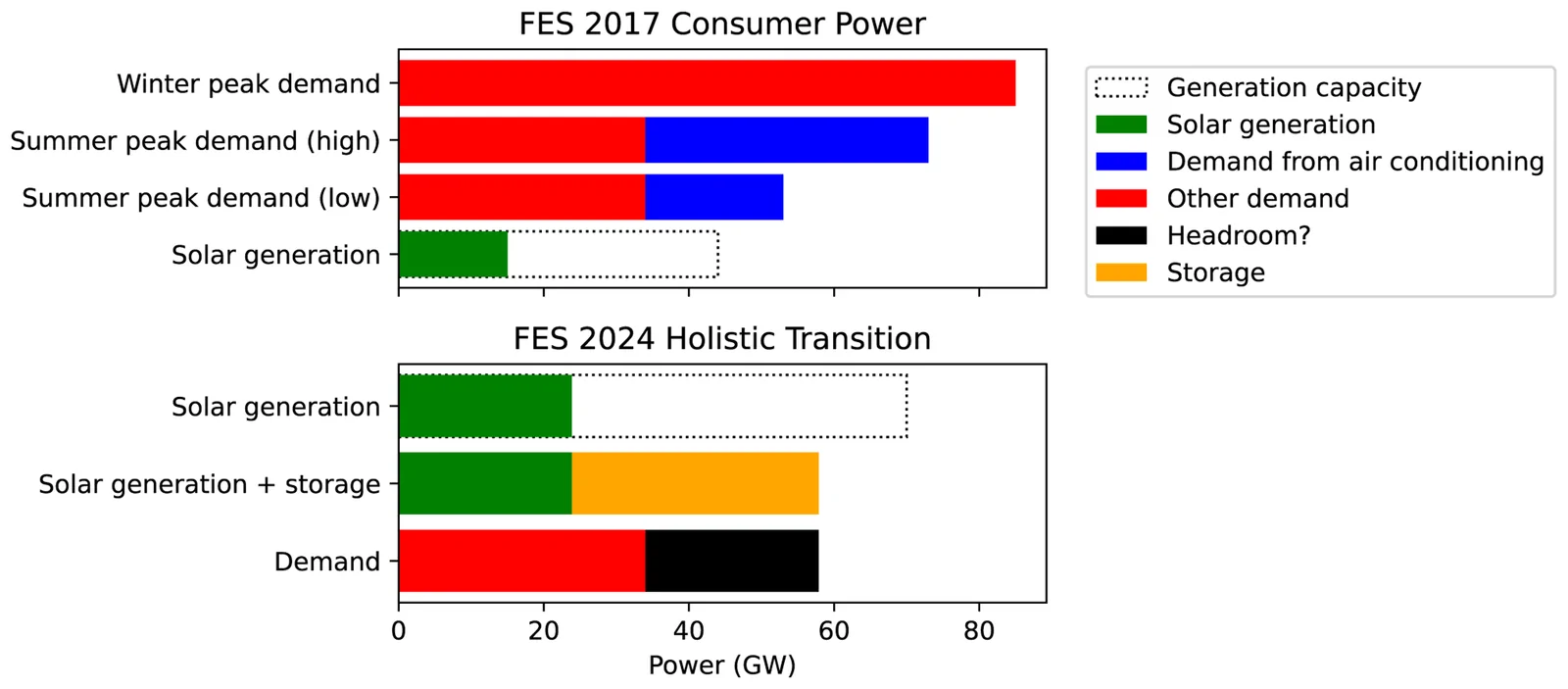
Demand and supply comparisons in Future Energy Scenarios.

A simple comparison of supply and demand was included in an earlier version of the FES, which we illustrate with Fig. 2. The FES 2017 had the “Consumer Power” scenario in which two thirds of GB homes would have air conditioning and global warming was 3 °C, peak demand from air conditioning was estimated at 39 GW contributing to a total peak demand of 53 to 79 GW. This scenario also had high peak demand contributions from electric vehicle charging. The relevant peak in demand in the scenario occurs at 5.30 PM on a summer weekend evening, at which time 44 GW of solar capacity was estimated to provide 15 GW of power (a 34% hourly capacity factor). Therefore, in this scenario, peak demand would exceed generation from renewables unless wind, storage, or other sources are available to make up the 38–64 GW difference.

The FES 2024 HT scenario has milestones of 70 GW of solar generation capacity by 2040, and total storage capacity (including vehicle-to-grid) of 34 GW by 2030 [12]. Under the assumptions from the FES 2017 Consumer Power scenario (previous paragraph), 70 GW of solar generation capacity at 34% capacity factor, and 34 GW from storage would mean at most 58 GW of demand could be met with renewables and storage combined. With 14 to 40 GW of other demands (again from the FES 2017 Consumer Power scenario), this would leave 18 to 44 GW of headroom for air conditioning, enough for 18 to 44 million portable air conditioning units, on the order of one per household. Storage capacity or other flexibility becomes very important for meeting air conditioning demand in the evening or night, which might also compete with demand for electric vehicle charging. This extremely rough, first principles calculation suggests that there could be sufficient generation capacity for a large proportion of households in GB to simultaneously run a portable air conditioner from renewable energy and storage. This is more sensitive to the amount of storage output capacity than the amount of solar generation capacity because of the low solar capacity factor.

A more detailed analysis would dynamically model variable renewable capacity factors, the storage level, hourly cooling demand and hourly profiles of other power demands. It will be important to consider weather conditions with low wind and high temperatures, and to consider future climate change. Hot, low wind days do not necessarily have completely clear skies. High cloud reducing solar supply was a factor in the August 2020 California incident discussed above. Demand side flexibility will also be important for determining peak loads.

Other factors that should be considered include efficiency of electricity generation and transmission. Transmission constraints may be important if sources of generation or storage are far away from centres of demand [64], and transmission capacity may be reduced by extremely hot weather [6]. Demand for air conditioning could have a different spatial distribution from that for heating, e.g. highly concentrated in southern cities. The spatial distribution of power supply may also be different in hot, low wind conditions compared to average conditions. The efficiency of power plants can also be reduced by high temperatures [6]; resource de-rating is a substantial proportion of the reserve margin calculation in many summer-peaking systems [72]. Reduced efficiency of power plants and of transmission contributed to the near-shortfall in electricity supply during the July 2022 heatwave in GB [6].

It may be important to model demands depending upon urban temperatures, noting that future projections of these are hotter, and even more uncertain, than ambient temperatures generally [73–75].

In the outages in California in 2020 [7], as well as in outages in Tokyo in 1987 [76], an exceptionally hot day led to record high electricity demand that was greater than forecast. In both cases, although high levels of demand from air conditioning were normal and planned for, demand was still underestimated in exceptional weather conditions by enough of a margin to create an operational incident in combination with other stresses. This demonstrates the importance of adequacy testing against less frequent, but higher system stress, exceptional weather conditions.

Finally, uncertainties in the design of the future system must be considered. Ideally the energy system will need to be secure and decarbonised in the long term. Beyond 2050 there is greater difference between climate scenarios and more uncertainty in climate projections, as well as more uncertainty over what the energy system will look like. Because peak summer DNR is unexamined in GB, system adequacy and resilience under high AC uptake remains untested.

### 4.3 Distribution capacity

Will distribution networks be able to deliver peak loads from air conditioning in GB? Delivering electric power for heating will require expansion and upgrades of the electricity transmission and distribution system to handle greater loads and transmit power from renewable generation sites [77]. Increasing demand from electric vehicles and heating seem to be a larger concern than space cooling [78,79]. The vastly larger scale of peak and total electricity consumption for heating suggests that transmission and distribution capacity could be sufficient for cooling.

However, distribution faults already occur at increased rates when daily maximum temperatures exceed 30 °C [6]. Risk assessments published by the distribution network operators (DNOs) consider risks to electricity distribution caused by the direct effect of high temperatures on their equipment, but also coincident high demand. In the risk assessment from one DNO, it is stated that this risk can be well mitigated as “demand is forecast using Distribution Future Energy Scenarios (DFES) and new transformers are installed based on projected load”, also noting that transformers currently operate with high margins of safety, but that standards may need to be reviewed in future [80]. While it may be concerning that the DFES contain similar assumptions about the growth of domestic air conditioning as the FES [81], DNOs appear to be aware of this [77]. Work commissioned by ENWL has sought to evaluate whether investments in reinforcement of distribution assets will need to be brought forward in response to cooling demand, and whether this can be mitigated by demand response for cooling; finding that growing cooling demand could cause some substations to need reinforcement sooner, substantially affecting network planning [77]. Small areas with high concentrations of air conditioning, or other temperature dependent demands, may still produce distribution issues at a local scale. Review of these policy documents suggests a stakeholder consensus that electrification of heating is a greater challenge than cooling for the distribution network, and that a distribution network sufficient for electrified heating and transport will be mostly sufficient for expanded cooling, but that fast growth of air conditioning could lead to reinforcement having to occur sooner and therefore the bringing forward of some costs.

While the future scenarios of growth of air conditioning used for distribution system planning are narrow, the comparison of scale between existing overall peaks in electricity demand and potential future summer peaks is more valid and suggests that distribution capacity should be sufficient for high residential penetration of air conditioning.

## 5 Cooling as an opportunity for mitigation-adaptation synergy

As the electricity system shifts toward renewable energy, a change in mindset around air conditioning may be required. Increased use of air conditioning may not necessarily mean that more fossil fuels are used, unlike in the current energy system, and may not mean that system costs are higher. This would present opportunities to improve adaptation to climate change and mitigation of emissions.

With the changing climate, more action is needed to address the health, wellbeing, and productivity effects of overheating buildings, and in some cases air conditioning will be the most cost-effective option. Current policy addressing overheating emphasises “passive first”, partly motivated by avoiding energy use. However, if avoiding energy use becomes less effective, then the focus could shift from efficiency to effectiveness. Passive strategies and air conditioning are not mutually exclusive, and passive strategies may in some cases provide comfort more effectively or adaptively than air conditioning and are more resilient to power failure. The coming shift in the energy system could be an opportunity for air conditioning to take a larger role in strategies to address overheating.

As the marginal system costs of electricity generation (and emissions intensity during the transitional period where unabated gas is used flexibly) will be greatest at times of high DNR in the future energy system, the priority for sustainable building design should shift from reducing overall energy use to reducing energy use at times of high DNR. Reducing overall peaks will remain important for reducing pressure on the transmission and distribution systems. Therefore, decreasing winter evening heating use will be crucial, whereas limiting summer daytime cooling use may have less relevance. For example, increasing the insulation or solar gains of a building may be worthwhile in terms of carbon and operational costs even if it leads to air conditioning use. Furthermore, methods that shift electricity consumption from cooling demand closer to the solar peak could be just as effective as avoiding cooling demand altogether.

Another opportunity sits at the intersection of climate mitigation and adaptation. In some cases, air-to-air heat pumps (AAHPs) are suitable as the main heating system of a home, presenting an opportunity for synergy between climate mitigation and adaptation policy [46]. AAHPs sold in the UK often have the ability to heat as well as cool. In certain buildings AAHPs are the most cost effective way to electrify heating, however, they had not been supported by policy which instead favours water-circulating heating systems [46]. The greatest reason for this lack of support was concern about encouraging the expansion of cooling, which would increase energy consumption and therefore fossil fuel use [46,82,83]. But, it is unclear if avoiding cooling should continue to be an objective, in which case it would no longer be justified to exclude AAHPs from support.

Rates of installation of heat pumps are off track, and the UK is behind comparable countries [21,84]. AAHPs may offer distinct advantages in some cases. Heating-dominated countries which have been successful in heat pump adoption have mainly used AAHPs rather than water-circulating systems [44,46], although there may be reasons they are less suitable in the UK [44,46]. The cooling provided by AAHPs offers added benefit to consumers: not only decarbonising their home heating but also improving their comfort in summer. Cooling could provide an additional pull for decarbonisation of heating if implemented properly, and could therefore accelerate the transition to decarbonised heating. [44,46,85] As domestic energy use in the UK is dominated by winter heating, this could be net beneficial in terms of greenhouse gas emissions if summer energy use in the near future will be generally low carbon [46]. This would depend upon air-to-air heat pumps replacing combustion heating, rather than only providing cooling.

Supporting air-to-air heat pumps for heating could simultaneously address climate adaptation and mitigation in an integrated and low-cost way, presenting a major policy opportunity. The co-benefits of greenhouse gas emissions mitigation actions have been highlighted as an important pathway to accelerate climate action [58]. Comfort cooling could be a direct co-benefit to households of replacing their fossil fuel heating with an AAHP. Fewer people being exposed to overheating and its health effects would be a public health co-benefit.

Recent changes in government policy have moved in a direction that supports AAHPs. Changes to the Boiler Upgrade Scheme announced in November 2025 [86] mean of AAHPs can now receive grant support, whereas previously only water-circulating heat pumps were supported, removing one barrier to their adoption. Amendments which came into force in May 2025 [87] allow for air-source heat pumps that provide both heating and cooling to be installed under permitted development rights (whereas previously planning permission was required), removing an administrative barrier to their deployment in existing buildings.

Electricity demand is not the only downside to air conditioning, and our aim is not to downplay these. The global warming potential of refrigerants will still be a concern [46]. Waste heat from air conditioners is emitted outdoors, raising the outdoor air temperature in dense urban areas [88]: worsening overheating for buildings that do not have air conditioning, reducing the efficiency of air conditioners, and directly affecting people outdoors. Furthermore, air conditioners are not resilient to power failures [5]. Some would argue that having air conditioning removes the stimulus that would drive physiological acclimatisation or adoption of more resilient cooling strategies. Air conditioners and the energy required to run them may be unaffordable to some, so a societal response relying on air conditioning could be inequitable, although air conditioning may be more affordable and practical than alternative solutions in some cases [89].

The assumption that air conditioning growth will be slow in the UK is affecting investment decisions being made now. The system could be committing to too little solar generation, storage, or flexible generation capacity to cover summer demand net of renewables under extreme conditions. The assumption that air conditioning growth needs to be limited contributed to AAHPs being excluded from direct and indirect support, which may be hampering the electrification of heating and increasing the costs of the transition.

## 6 Conclusion

In summary, domestic air conditioning ownership may be growing quickly, potentially invalidating the assumptions used in energy system planning. However, GB and other temperate countries are investing in capacity to meet electrified winter heating demands and electric vehicle charging with renewables and it is unclear whether additional residential cooling demand would require additional infrastructure and or lead to additional positive DNR. Therefore, the effect of air conditioning demand on system adequacy merits greater attention. Looking at how air conditioning has been adopted in other countries can be informative but treating air conditioning ownership as exogenously driven by temperature based on data from other countries, or as having a characteristic timeframe, may give false confidence in the predictability of the future trajectory. Rather than focussing on what the most likely future trajectory of air conditioning uptake may be, we recommend that a range of plausible scenarios are considered, with greater attention to quantifying potential trade-offs which we have shown are currently neglected in electricity system planning in GB.

While other problems with air conditioning should not be forgotten, if the electricity system trade-offs presented by air conditioning are weak in the GB context, then air conditioning could play a greater strategic role in climate mitigation and adaptation. There is a risk that we build the electricity system around unrealistically low cooling demand assumptions, leading to missed decarbonisation targets or greater operational challenges, but also a risk that we incorrectly treat assumptions of low demand as constraints and therefore miss the opportunities that air conditioning might provide.

Cooling represents a blind spot in GB energy system planning. Quantitative, robust analysis of the sensitivity of electricity system designs in temperate countries (including generation, storage and flexibility, transmission, and distribution) to air conditioning demand levels could close the gap. This should include the effects of co-occurring weather conditions, and the correlations of supply and demand at daily and hourly level (as opposed to merely seasonal level). This may be supported by consideration of a wider range of future scenarios, informed by international air conditioning ownership data, consumer behaviour, electricity systems data, and smart meter. Without considering cooling, we risk designing an energy system that is unprepared for the realities of a hotter, more electrified future, missing both critical vulnerabilities and transformative opportunities.

## Figures and Tables

**Table 1:** Estimates of proportion of homes with air conditioning from various sources [13,17,24–26].

Publication	Proportion with air conditioning	Notes	Survey year	Source	Scope
Energy Follow Up Survey, DECC/BEIS/DESNZ [26]	2.8%	Of which 71% portable air conditioning	2011	Representative survey *(n =* 2616)	Homes in England
Energy Follow Up Survey, DECC/BEIS/DESNZ [26]	2.3%	Of which 70% portable air conditioning	2017	Representative survey (*n* = 2528–2632)	Homes in England
English Housing Survey, MHCLG [24]	3.1%		May 2021-March 2022	Representative survey (*n* = 10,547)	Homes in England
“Cooling in the UK” BEIS/DESNZ [13]	3-5%	Portable units may be underestimated	2021	Estimate by industry group (BSRIA)	Homes in the UK
“A nation unprepared: Extreme heat and the need for adaptation in the United Kingdom” Khosravi et al. [47]	19%	Of which 81% purchased 2022-2023	2023	Representative survey (*n* = 1580)	Homes in GB
“Project Cooldown”, Impact Research Ltd. and ENWL [77]	8%	Of which 69% portable air conditioning	2024	Opt-in survey panel (*n* = 1000)	UK households

**Table 2:** Estimates of air conditioning ownership growth rates collated by the Tyndall Centre [33] with additional primary data.

Country				Annualised relative growth	Years to increase 2×	Source
USA	Year	1960	2001			McLachlan et al. [33]
	Ownership	12%	81%	4.7%	15	
Australia	Year	1994	2014			McLachlan et al. [33]
	Ownership	32.5%	74%	4.1%	17	
France	Year	2000	2010			McLachlan et al. [33]
	Ownership	0.5%	5%	23.0%	3	
Austria	Year	2019	2021			
	Ownership	5.29%	7.81%	19.4%	4	Statistics Austria [34]
Belgium	Year	2016	2022			Statbel [27]
	Ownership	9%	18%	11.6%	6	
Bulgaria	Year	2008	2023			NSI [35]
	Ownership	13%	61%	10.3%	7	
Czechia	Year	2015	2021			
	Ownership	1%	4%	20.7%	3	Czech Statistical Office [36]
Spain	Year	2003	2019			AIMC [37]
	Ownership	14%	38%	6.5%	11	
Hungary	Year	2010	2020			STADAT [38]
	Ownership	4%	14%	12.3%	6	
Italy	Year	2001	2022			ISTAT [39]
	Ownership	11%	51%	7.4%	9	
Portugal	Year	1995	2015			PORDATA [40]
	Ownership	2%	16%	9.6%	7	
Slovenia	Year	2012	2022			Statistical Office of the Republic of Slovenia [41]
	Ownership	16%	38%	8.4%	8	

## Data Availability

Data will be made available on request.
